# Survival rate of gastric cancer in Iran; a systematic review and meta-analysis

**Published:** 2016

**Authors:** Yousef Veisani, Ali Delpisheh

**Affiliations:** 1 *Psychosocial Injuries Research Center, Ilam University of Medical Sciences, Ilam, Iran*; 2 *Department of Clinical Epidemiology, Ilam University of Medical Sciences, Ilam, Iran *

**Keywords:** Gastric cancer, Survival rate, Meta-analysis, Systematic review, Heterogeneityndrial

## Abstract

**Aim:**

In this study, we aimed to estimate one- to five-year survival rates in Iranian patients with gastric cancer (GC). In addition, we preformed subgroup analyses and meta-regression to explore possible sources of heterogeneity between studies.

**Background:**

According to literatures, there has been increasing attention to the long-term survival rate in patients with GC in Iran. However, results have been inconsistent and remain controversial in overall survival rates.

**Patients and methods:**

Literature searches were conducted using PubMed, Scopus, and ISI, as well as Magiran, Medlib, SID, and Iran Medex databases. Studies were pooled and summary one to five survival rates were calculated. Univariate and multivariate regression analyses were used to explore possible sources of heterogeneity among studies. Subgroup analyses were also conducted. Analyses were conducted using the STATA statistical software package.

**Results:**

Final analysis of 29361 patients from 26 eligible studies was performed. The overall survival rate (one to five years) in all studies, by meta-analysis of 24, 14, 23, 12 and 22 studies were 52%, 31%, 24%, 22%, and 15%, respectively. Meta-regression analysis showed an increase in one- and five-year survival rate over the time (Reg Coef = 0.016, p= 0.04) and (Reg Coef= 0.021, p= 0.049), respectively. Positive heterogeneity was observed between quality of papers and data sources (P<0.001).

**Conclusion:**

More than half of GC deaths happened in the first year at diagnosis, and another 30% plus they occurred during the second year after confirmed diagnosis. Our results admit lower survival rates in Iran, similar to other developing countries.

## Introduction

 Every year, 7 million lives are lost due to preventable and treatable cancers (1). Incidence rates of cancers could increase substantially in the future, with up to 15 million new cases in 2020, most of which will be in developing countries (2). Among all cancer types, gastric cancer (GC) is one of the leading causes of death in recent decades, and its death toll has been steadily increasing in Asia and across the globe (3). Environmental factors such as obesity and gastroesophageal reflux disease are thought to contribute to its deadliness (4). At diagnosis, 23% of GC cases are localized, 32% are detected in the lymph nodes, and 34% are metastatic (5). In localized cases, treatment usually involves a combination of surgery, radiation, and chemotherapy, supplemented with adjuvant therapy (6, 7).

Misdiagnosis is the greatest obstacle facing treatment of GC in Iran (8-10). In most cases, by the time the disease is detected and properly diagnosed, surgery is the only viable option. Therefore, early detection and screening programs are critical to improve prognoses in GC patients (11, 12). In the last decade, there are a growing number of Iranian studies, which have focused on the rate of GC survival. Results of these studies indicate that survival rates in Iran are consistent with those in other developing countries, but lower than those in developed countries (13). 

It is difficult to conduct population-based cancer studies in Iran, due to incomplete hospital records, careless registration processes, insufficient training, haphazard patient follow-up policies, and a lack of regional and provincial cancer centers. Inconsistent GC survival rates are also a prevalent feature in Iranian medicine, the lowest and highest of which were 81% (14) and 21% (15), respectively, for one year, and 31% (16) and 5.4% (17), respectively, for five years. 

**Figure 1:**
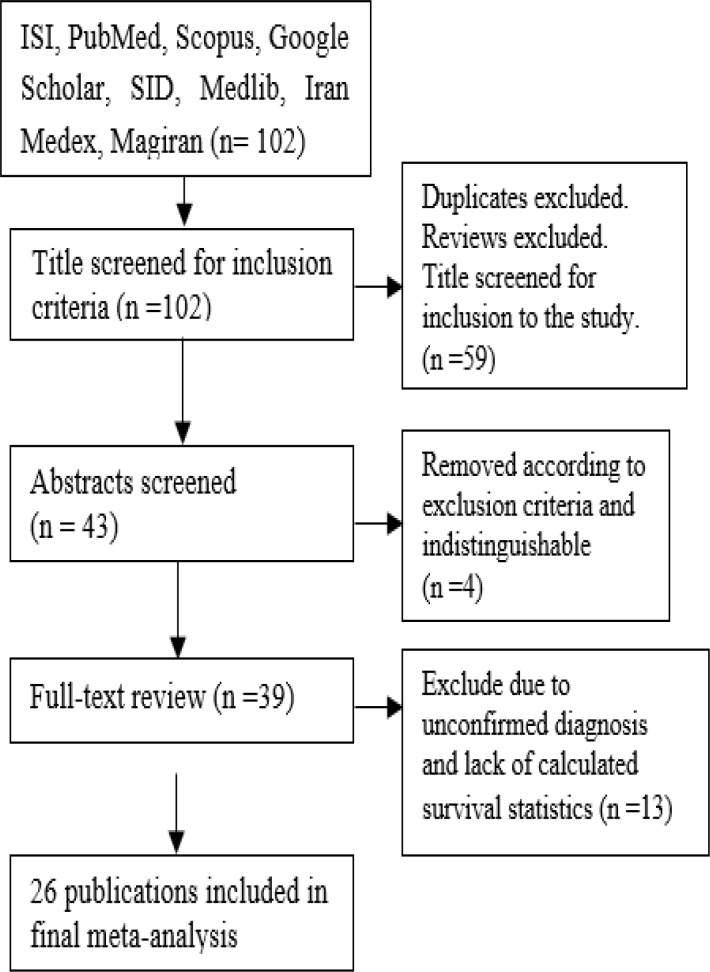
Flow diagram shows different steps involved in searching for relevant publications (2005–2015

According to our research, no recent systematic review has focused on GC survival in Iran. The present study intends to contribute to the extant literature by providing a systematic review and meta-analysis of one- and five-year survival rates in Iranian GC patients, and performing subgroup analyses and meta-regression to explore possible sources of heterogeneity among included studies. 

## Methods

Through an electronic and manual search, 102 papers were identified (Table1). After exclusion of reviews and duplicate articles, as well as title screen, 43 separate publications remained for further appraisal. Remained articles, subsequent to abstract and full text review we removed 17 publications according to exclusion criteria and in final data set consisted of 26 publications (Figure 1). Interpretation results of table 2 shows attention to survival rate in patients with GC in Iran is increasing as if (64.4%) of final articles were published in 2011 onwards. Hospital records were the primary data source for the greater part of studies (61.5%). The pooled participants in the study were 29361 patients with GC (Table1). 

Twenty-four eligible papers including 28949 patients were included in meta-analysis to estimate of one-year survival rate. The overall one year survival rate in patients with GC in Iran was 0.52 (95% CI: 0.52 to 0.53). A significant heterogeneity among these studies was observed (heterogeneity statistic= 379.79, P<.001, I^2^= 98.5%, 95% CI 97.4–99.8) (Figure 2). 

For Meta-analysis of five-year survival rate twenty-two publications with 28268 patients were considered. The overall five year survival rate in patients with GC in Iran was 0.15 (95% CI: 0.15 to 0.16).

**Table 1:** Feature and characteristic studies included in study

Ref. No.	First Author (Year of Pub)	Years of flow Sitting	No. of Patients	Data source	Analysis	Survival Rate (%)					Quality*
						1-Year	2-Year	3-Year	4-Year	5-Year	
(19)	Biglarian A 2011	2002-2007, Tehran	436	Hospital records	Cox proportional hazards	78	53	41	32	17	High
(20)	Mehrabian AA 2010	2001-2006, National	19537	Iran Cancer Registration Center	Life time table	49	29	23	23	15	High
(16)	Soroush A 2013	2008-2010,	98	Hospital records	Kaplan–Meier method and Cox proportional hazard models	60				31	High
(21)	Zare A 2013	1995-1999, National	330	Iran Cancer Institute	Cox proportional hazards model	66	42	31	26	21	High
(22)	Baghestani AR 2009	2003-2008, Tehran	178	Hospital records	Bayesian Weibull and Exponential models	80	52	35			High
(23)	Moghimi-Dehkordi B 2008	2001-2006, Tehran	746	Cancer Registry Center	life-table method and Wilcoxon (Gehan) test	73	50	40	33	29	High
(24)	Samadi F 2007	2000-2004, Ardabil	279	Hospital records	Kaplan–Meier method and Cox proportional hazard models	41				8	High
(25)	Noorkojuri H 2013	2003-2008, Tehean	216	Tehran Cancer Registry	Cox proportional hazards and smoothing methods	80	56	40	35	30	High
(26)	Yazdani-Charati J 2014	2007-2010, Sari	190	Hospital records	Kaplan-Meier method	45	26	8			High
(27)	Ghadimi Gh 2011	1990-199, Babol	484	Cancer Registration Center	Weibull, Log-normal, and the Log-logistic model	24		16		15	High
(28)	Maroufizadeh S 2011	2003-1008, Tehean	213	Cancer Registration Center	Cox and Additive hazards models	79		35		14	High
(15)	Bashash M, 2011	2004, Ardabil	261	population-based cancer registries	Life-tables	21					High
(29)	Movahedi M, 2009	2001-2005, National	3189	national cancer registry	Kaplan-Meier method	48	27	19	16	13	High
(17)	Veisani Y, 2013	2006-2011, Sanandaj	239	Hospital records	Kaplan-Meier method	41	17	13	10	6	High
(30)	Atoof F, 2010	1995-2004, Tehran	330	Hospital records	Kaplan-Meier and Weibull Cure Models			32		20	Medium
(31)	Roshanaei Gh, 2012	2003 – 2007, Tehran	400	Hospital records	Cox proportional hazards	74	54	31	26	23	Medium
(32)	Moghimi-Dehkordi B, 2007	2001-2005, Tehran	442	Cancer Registration Center	Kaplan–Meier and Cox proportional hazard models	54	30	24	18	16	Medium
(33)	Barfei F, 2014	2007-2008, Tehran	99	Hospital records	Kaplan–Meier and Cox proportional hazard models	59		40		18	Low
(34)	Kashani H, 2011	1995-1999, Tehran	330	Hospital records	Kaplan–Meier and Cox proportional hazard models	62	41	31	24	20	Medium
(35)	Baeradeh NA, 2015	2006-2010, Yazd	136	Hospital records	Kaplan–Meier and Cox proportional hazard models	61	45	31	26	25	Medium
(36)	Zeraati H, 2005	1995-1999, Tehran	129	Hospital records	A non-homogenous semi-Markovian stochastic process	67		31		19	Medium
(37)	Ghorbani S, 2013	2007-2012, Sari	430	Cancer Registration Center	Kaplan - Meier and univariate analysis	64	44	34	28	19	Medium
(14)	Roshanaei Gh, 2010	2003-2007, Tehran	262	Hospital records	Kaplan–Meier and Cox proportional hazard models	81		45		30	Low
(38)	Roshanaei Gh, 2011	2003-2007, Tehran	93	Hospital records	Kaplan–Meier models	42		19		13	Medium
(39)	Larizadeh MH,2013	2003-2011-Kerman	82	Hospital records	Kaplan-Meier methods			53		22	Low
(40)	Gohari MR, 2014	2002-2007, Tehran	232	Hospital records	Kaplan-Meier methods	77		26			Low

**Table 2:** Subcategories analysis of one to five survival rates by quality and data source

**Subcategories**		**Survival Rate% (95% CI)**					**Heterogeneity**	
		1-Year	2-Year	3-Year	4-Year	5-Year	I^2 (^%)	P value
Quality	High	51(50-51)	30(29-30)	23(22-23)	22(22-23)	15(14-15)	98.8	<0.0001
	Medium	63(61-65)	42(39-44)	29(28-31)	24(22-26)	19(18-21)	89.6	<0.0001
	Low	77(73-80)	-	38(34-41)	22(13-31)	26(21-30)	87.5	<0.0001
Data Source	Hospital records	67(65-68)	41(38-43)	27(26-29)	22(20-24)	16(14-17)	96.7	<0.0001
	Cancer registry center	50(49-51)	30(29-30)	23(23-24)	22(22-23)	15(15-16)	97.7	<0.0001
Overall survival rate		52(52-53)	31(30-31)	24(23-24)	22(22-23)	15(15-16)	95.6	<0.0001

**Figure 3:**
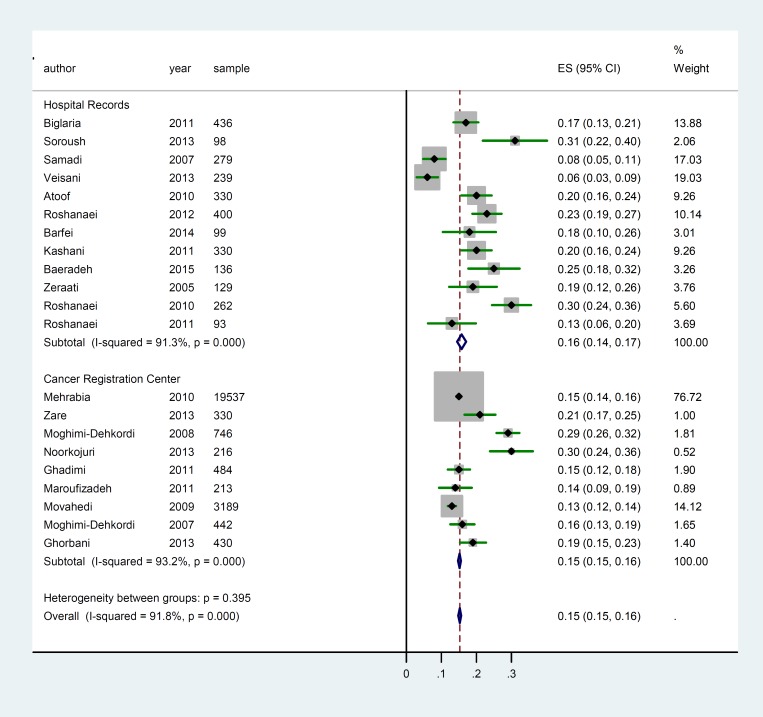
Meta-analysis of the five year survival rate by different data source (Hospital Records and Cancer Registry Centers)

**Figure 4:**
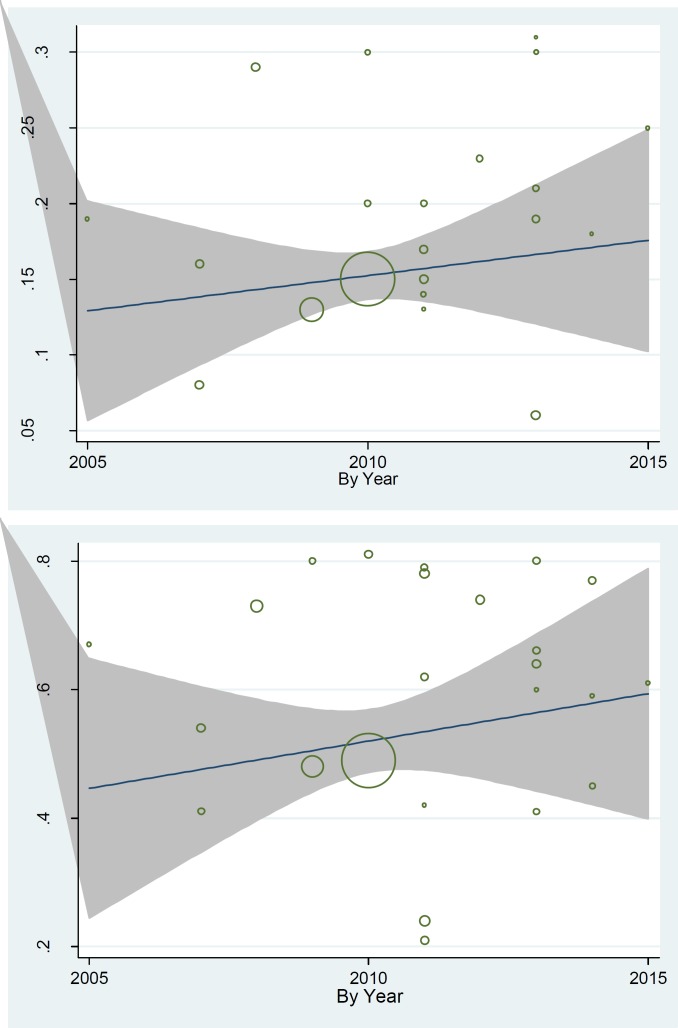
Meta-regression plots of change in one and five survival rate according to changes in continuous study moderator’s year

A significant heterogeneity among these studies was observed (heterogeneity statistic= 472.23, P<0.001, I ^2^ = 91.8% (95% CI: 90.1–93.5) (Fig. 3). Subgroup analysis was preformed to explore possible sources of heterogeneity among studies. Results of subgroup analysis showed a positive heterogeneity between quality of papers and data sources (P< 0.001). Table 2 presented these results; one- to five-year survival rate in publication with good quality is lower than articles with medium and low quality, respectively. Also one- to five-year survival rate in studies with cancer registry center data source is lower than hospital records. Results of meta-regression showed an association between publication year and one year survival rate, as well as five-year survival rate. Thus, year of publication is one of the main causes of variability in results of one- to five-year survival rate (Reg Coef= 0.016, p= 0.04) and (Reg Coef= 0.021, p= 0.049), respectively (Fig. 4). According to results, an increasing rate of survival was observed across the study period. Also, we examined sample size as another explanatory factor to variability in results, which showed sample size was another reason for this inconsistency in results (Reg Coef= 0.00033, p= 0.027). Studies with a large sample size had a lower survival rate compared to studies with small sample size. 

## Results

Using electronic searches, 102 papers were identified. After exclusion of reviews and duplicate articles and title screen, 43 separate publications remained for further appraisal. Remained articles, subsequent to abstract and full text review, we removed 17 publications according to the exclusion criteria and in the final data set consisted of 26 publications (Fig. 1). Interpretation results of table 2 shows attention to survival rate in patients with GC in Iran is increasing as if (64.4%) of final articles were published in 2011onwards. Primary data source for the greater part of studies (61.5) was hospital records. 

The pooled participants in study were 29361 patients with GC (Table 1). 

Twenty-four eligible papers, including 28949 patients were included in meta-analysis to estimate one-year survival rate. The overall one-year survival rate in patients with GC in Iran was 0.52 (95% CI: 0.52 to 0.53). There was a significant heterogeneity among these studies (heterogeneity statistic = 379.79, P<0.001, I ^2^ = 98.5%, 95% CI 97.4–99.8) (Fig. 2). 

For Meta-analysis of five-year survival rate, we considered twenty-two publications with 28268 patients. The overall five year survival rate in patients with GC in Iran was 0.15 (95% CI, 0.15 to 0.16). A significant heterogeneity among these studies was observed (heterogeneity statistic= 472.23, P<0.001, I ^2^= 91.8% (95% CI: 90.1–93.5) (Fig. 3). Subgroup analysis was performed to explore possible sources of heterogeneity among studies. Results of subgroup analysis showed a positive heterogeneity between quality of papers and data sources (P<0.001). Table 2 presented these results; one- to five-year survival rate in publication with good quality is lower than articles with medium and low quality, respectively. Also, one- to five- year survival rate in studies with cancer registry center data source is lower than hospital records. Results of meta-regression showed an association between publication year and one-year survival rate, as well as five-year survival rate. Thus, year of publication is a cause of variability in results of one and five year survival rate (Reg Coef= 0.016, p= 0.04) and (Reg Coef= 0.021, p= 0.049), respectively (Fig. 4). According to results, an increasing survival rate across the study period was observed. Also, we examined the sample size as other explanatory factor to variability in results and results showed sample size was another reason for this inconsistency in results (Reg Coef= 0.00033, p= 0.027). Studies with larger sample size had a lower survival rate.

## Discussion

In the present meta-analysis, we employed a large sample size to generate a reliable estimation of GC patient survival rates. We found a significant heterogeneity in our results, sources of which we explored using meta-regression and stratified subgroup analysis according to characteristics of the included studies. Our results showed that sample size and publication year were significant contributing factors to heterogeneity, where larger sample size and later year of publication were associated with a lower recorded rate of survival. In the present study, heterogeneity might result from different characteristics of patients, differing stages of disease progression, adjuvant treatment, duration of patient follow-up, or histological type. Due to limited resources in previous studies, we were unable to specify the role of these features that might contribute to disparate survival rates.

Ultimately, 26 studies were incorporated into our meta-analysis. Estimation of overall survival rate, and the one- through five-year rates of 24, 14, 23, 12, and 22 were 52%, 31%, 24%, 22%, and 15%, respectively. These numbers indicate that more than half of GC deaths occurred within the first year following diagnosis, and another 30% took place during the second year. It is the greatest difficulty about patients with GC in Iran versus worthwhile clinical finding to correct rational strategies to address these problems. Various histological and demographic factors such as age, gender, surgery and treatment type, cancer site, grade of tumor, as well as metastasis have been found to impact the rate of GC survival in Iran. An investigation into risk factors associated with GC may help to reduce the probability of death in patients. Other strategies include a comprehensive follow-up plan for patients with premature signs of the disease, as well as the study and application of suitable treatments (5).

Results of the present study suggest that the overall five-year survival rate in Iran (15%) is lower than the survival rate in countries such as China (29.6%), the United States (37%), Switzerland (22%), France (30%), and Japan (40-60%)(1, 13, 41). We classify possible explanations for this inconsistency into three main factors: cancer stage at diagnosis, patient characteristics, and treatment process. In most studies, late diagnosis was related to a lower survival rate, while diagnosis at an early stage was determined to be the most important predictive factor for survival. In terms of patient characteristics, a lower survival rate was associated with older and socioeconomically disadvantaged patients, as well as those who did not respond favorably to the treatment. Finally, mixed therapies incorporating chemotherapy, radiotherapy, and surgery were shown to strongly enhance the rate of survival.

As mentioned, a nationwide lack of cancer registry centers presents an additional obstacle to investigating GC in Iran, and the results of this study demonstrate this limitation. Although 64.4% of sources were derived from cancer registry data, the overall survival rates were lower than the results obtained from hospital records. Hospital records in developing countries are generally limited because data are missing, riddled with errors, or not deliberately gathered for the purpose of later scrutiny. Additionally, eligible patients for the present study were from one distinct hospital and could not represent all coverage patients in a population.

The present meta-analysis expanded the number of participants to produce reliable and generalizable results regarding the GC survival rate in Iran. Limitations of this study were heterogeneity among 26 included studies, a scarcity of abstracts, and the inclusion of only 11 studies out of 102 gathered due to lack of available survival statistics. These limitations could impact the findings of this analysis. 

According to our research, more than half of GC deaths occurred in the first year after diagnosis, and another 30% took place during the second year. These findings support previous reports that suggest a poor diagnosis is the greatest challenge to GC patients in Iran.

## Note

**(**Please cite as:** Yousef Veisani, Ali Delpisheh. Survival rate of gastric cancer in Iran; a systematic review and meta-analysis. Gastroenterol Hepatol ****Bed Bench**** 2016;9(2):78-86).**
